# High-performing physicians are more likely to participate in a research study: findings from a quality improvement study

**DOI:** 10.1186/s12874-019-0809-6

**Published:** 2019-08-07

**Authors:** Simone Dahrouge, Catherine Deri Armstrong, William Hogg, Jatinderpreet Singh, Clare Liddy

**Affiliations:** 1 CT Lamont Primary Health Care Research Centre, Bruyère Research Institute, 113-43, rue Bruyère St, K1N 5C7, Annex E, Ottawa, ON Canada; 2 Department of Family Medicine, University of Ottawa, Ottawa, ON Canada; 3 Department of Economics, University of Ottawa, Ottawa, ON Canada; 4 The Institute for Clinical evaluative Sciences, Ottawa, ON Canada

**Keywords:** Volunteer Bias, Quality improvement, Research methods, Primary care

## Abstract

**Background:**

Participants in voluntary research present a different demographic profile than those who choose not to participate, affecting the generalizability of many studies. Efforts to evaluate these differences have faced challenges, as little information is available from non-participants. Leveraging data from a recent randomized controlled trial that used health administrative databases in a jurisdiction with universal medical coverage, we sought to compare the quality of care provided by participating and non-participating physicians prior to the program’s implementation in order to assess whether participating physicians provided a higher baseline quality of care.

**Methods:**

We conducted clustered regression analyses of baseline data from provincial health administrative databases. Participants included all family physicians who were eligible to participate in the Improved Delivery of Cardiovascular Care (IDOCC) project, a quality improvement project rolled out in a geographically defined region in Ontario (Canada) between 2008 and 2011. We assessed 14 performance indicators representing measures of access, continuity, and recommended care for cancer screening and chronic disease management.

**Results:**

In unadjusted and patient-adjusted models, patients of IDOCC-participating physicians had higher continuity scores at the provider (Odds Ratio (OR) [95% confidence interval]: 1.06 [1.03–1.09]) and practice (1.06 [1.04–1.08]) level, lower risk of emergency room visits (Rate Ratio (RR): 0.93 [0.88–0.97]) and hospitalizations (RR:0.87 [0.77–0.99]), and were more likely to have received recommended diabetes tests (OR: 1.25 [1.06–1.49]) and cancer screening for cervical cancer (OR: 1.32 [1.08–1.61] and breast cancer (OR: 1.32 [1.19–1.46]) than patients of non-participating physicians. Some indicators remained statistically significant in the model after adjusting for provider factors.

**Conclusions:**

Our study demonstrated a participation bias for several quality indicators. Physician characteristics can explain some of these differences. Other underlying physician or practice attributes also influence interest in participating in quality improvement initiatives and existing quality levels. The standard for addressing participation bias by controlling for basic physician and practice level variables is inadequate for ensuring that results are generalizable to primary care providers and practices.

**Electronic supplementary material:**

The online version of this article (10.1186/s12874-019-0809-6) contains supplementary material, which is available to authorized users.

## Background

Voluntary participation is a hallmark of almost all research studies. This approach to recruiting participants can lead to the situation where those enrolling are different from those who refuse to participate. It is important to understand the differences between the participating and non-participating groups to evaluate the generalizability and relevance of the conclusions from the study. Comparisons of the groups are typically very limited because very little information is available from those who chose not to participate. Investigators must respect the individual’s decision to not participate and cannot survey them to collect data that would extend comparisons with those who agreed to participate. Publically available information is often used to show there are differences between groups, but these comparisons are typically limited to simple demographic information. [1] This is particularly problematic when information is unavailable that would characterize the participants who could benefit the most from the intervention. In many situations the physicians who most need the help are the least likely to participate; thus, the conclusions of the studies may not apply to them. [2]

There is evidence that family physicians who agree to participate in research are different from those who do not. Participants are more likely to be female, [3] younger, [3, 4] and graduates of local (as opposed to international) medical schools. [1] They are more likely to practice in rural areas, [4] under a salaried model, in larger practices, and to spend more hours per week providing direct patient care. [5] It has long been suspected that low performing physicians are less likely to volunteer to participate in studies. However, because studies only have access to recruitment data for non-participants, our current knowledge is limited to what can be discerned from readily available practice and provider recruitment information.

To explore this issue, we drew on data from the Improved Delivery of Cardiovascular Care (IDOCC) study, a pragmatic voluntary regional quality improvement (QI) research initiative conducted between 2008 and 2010 aiming to improve the delivery of cardiovascular care. Using data from population-based administrative health databases, we sought to compare the quality of care provided by participating and non-participating physicians prior to the IDOCC program’s implementation in order to assess whether the quality of care of physicians who participated in that QI project was higher than for those who did not participate. We considered using 14 well-established quality of care outcomes that capture cancer screening, access, continuity, and chronic disease management. [6, 7]

## Methods

### The IDOCC study

IDOCC was a stepped wedge, multifaceted, pragmatic, QI research study designed to assist primary care providers to improve their delivery of evidence-based care for the secondary prevention of cardiovascular disease. [8, 9] The trial was retrospectively registered on Clinicaltrials.gov on December 17, 2007 (https://clinicaltrials.gov/ct2/show/NCT00574808?id=NCT00574808&rank=1).

### Context and design

We conducted a cross sectional evaluation of baseline data for the IDOCC project, a voluntary QI research initiative in the Champlain Local Health Integration Network (LHIN), Ontario, Canada. Care of that population of 1.2 million is covered by a tax-supported universal health care system.

### Recruitment

The program was open to active family physicians providing general services within the Champlain LHIN in Eastern Ontario. Recruitment followed the Dillman approach [10] with invitation letters explaining the expectations from the participants, the role of the practice facilitator, the potential benefit to patients, and the eligibility of participants to claim continuing professional development credits. No financial compensation or any other incentives were provided.

We made every effort to reach all primary care practices within the region. We relied on various physician listings and a snowball approach to identify all potentially eligible family practices. Recruitment efforts were extensive, and were discontinued only after reaching the target number of 30 practices in each sub-region or until resources were exhausted. Recruitment letters were sent to the 533 primary care practices identified in the Champlain LHIN, inviting all eligible physicians to participate. Eligibility was restricted to practices that had been operating for at least 2 years so that adequate data was available for the indicator measurements, and to family physicians who provided comprehensive primary care to a panel of patients and were not planning to retire within the two-year intervention timeframe. Family physicians agreeing to participate signed an informed consent form. That form included permission to access their provincial health administrative data held at the Institute for Clinical Evaluative Sciences (ICES).

Ninety-nine practices were excluded due to non-eligibility (not providing comprehensive care, providing walk in services only or having been in place less than 2 years). Of the 434 eligible practices, 93, including 194 physicians, agreed to participate. Ten practices withdrew prior to the initiation of the intervention, primarily because of competing priorities, leaving 83 participating practices (19%) and 182 physicians.

### Dataset creation

We relied on data housed at ICES, a not for profit research institute that holds and links several health related billing databases for Ontario from which patient and physician profiles and performance measures can be obtained. Encounters with the healthcare system for all LHIN residents are captured in these databases, with the exception of those taking place in Community Health Centres (CHCs), a salaried model in which family physicians do not bill for services. The 35 participating CHC physicians were excluded from the evaluation, leaving 147 eligible physicians. The datasets were linked using unique encoded identifiers and analyzed at ICES.

To the extent possible, the health administrative dataset was created to mirror the eligibility criteria imposed in the study. The data set from this study is held securely in coded form at ICES. While data sharing agreements prohibit ICES from making the data set publicly available, access may be granted to those who meet pre-specified criteria for confidential access, available at www.ices.on.ca/DAS. The authors did not have special access privileges. Interested researchers would be able to access the data in the same manner as the authors*.*

#### Physicians

Target population (see Table 1): We identified all physicians who practiced within the Champlain LHIN using the practice postal code information contained in the ICES Physician Database (IPDB). We then applied the same exclusion criteria to those physicians as would have been applied in the intervention study. We excluded physicians who were recorded as having a specialty other than “General Practice” or “Family Practice with Emergency Medicine” in the IPDB. Applying that criteria at the IDOCC start date (April 2007) identified 1340 physicians: 1201 IDOCC non-participants and 139 IDOCC participants. We extended the eligibility years to 2009 to ensure that young physicians having participated in IDOCC could be captured, which identified an additional 182 physicians, including the remaining 8 IDOCC physicians who engaged in Step III (April 2009).

**Table 1:** Cohort Creation The number of physicians assessed at each eligibility step and in each group

Inclusion Criteria	Total eligible	IDOCC	Non-IDOCC
All physicians	3475		
Family physician	1522	147	1375
*Those identified in 2007*	*1340*	*139*	*1201*
*Those added in 2009*	*182*	*8*	*174*
Providing comprehensive care	1122	144	978
Caring for at least 100 patients	**1028**	**144**	**884**

Physicians meeting the inclusion criteria at each step

Family physician: All physicians with recorded specialty in the ICES Physician Database (IPDB) other than General Practice, Community Medicine, or Family Practice with Emergency Medicine are excluded

Providing comprehensive care: To limit the dataset to general practitioners, we excluded individuals whose billing pattern showed a specialized practice. We evaluated 21 comprehensiveness primary care fee schedule codes, and required that the physician had billed at least 8 of these codes over the past 2 years

Caring for at least 100 patients: We also excluded physicians to whom fewer than 100 patients could be attributed as these were likely to new graduates having not yet established their practice. The Ontario Ministry of Health and Long Term Care provides new graduates a fixed income during 48 months to allow them to establish their practice without financial hardship. These physicians are therefore not required to bill their encounters and could therefore not be assessed. Three IDOCC participants fell into that category

We limited the dataset to physicians whose billing patterns demonstrated that they provided general, comprehensive primary care (having billed at least eight of the 21 comprehensive primary care fee schedule codes during the past 2 years). This led to the exclusion of 400 physicians, including three IDOCC physicians who were new graduates working under capitation remuneration and thus offered a fixed income, independent of clinical activity, for 2 years while they established a practice panel. During this “income stabilization” period, physicians do not bill for their activities and would therefore not meet that criterion. While we know that these three physicians did provide comprehensive care, they were excluded to ensure the same criteria were applied to the two both groups.

In Ontario, the median patient panel size is approximately 1,400. We excluded physicians with fewer than 100 patients because these outliers likely provide walk-in services only, were near retirement or were new graduates who haven’t yet built their panel, and had too few patients to be adequately evaluated.

Time Period: Baseline characteristics were captured for the 2 years prior to the IDOCC April 2007 recruitment start date (April 2005–March 2007) for all physicians having met the criteria on that date. For the physicians identified in 2009, the baseline characteristics were evaluated for the two prior years (April 2007–March 2009).

Physician profile: We used the Corporate Provider Database and the IPDB to obtain information on physician characteristics, including sex, age, having received medical training abroad, remuneration (fee-for-service vs capitation) and team (non-interprofessional vs. interprofessional) structure, practice rurality, and practice group size at the end of the two-year period.

#### Patients

Patients officially registered to a physician were identified using the Client Agency Program Enrolment database and were attributed to that physician. Approximately 20% of the population is not formally registered to the family physician from whom they receive care. These individuals were “virtually” attributed to the family physician who provided the largest dollar amount of services over the baseline two-year period. [11, 12] Information on patient characteristics including age, sex, and rurality were drawn from Ontario’s Registered Persons Database. Income quintile was derived from the patients’ postal code using aggregate census data. Patients who acquired Ontario Health Insurance Program (OHIP) coverage at a time other than birth were deemed to be immigrants.

#### Quality of care measures

We considered 14 quality of care outcomes spanning four categories (cancer screening, access, continuity, and chronic disease management). These indicators represent measures widely accepted as reflecting primary care performance and are commonly used for that purpose). [6, 7] The cancer screening outcomes captured whether or not patients had undergone the screening test for which they were eligible (Ontario Breast Screening Program, OHIP laboratory fees). Access outcomes captured the rate of emergency room visits per year, both overall and for low urgency (National Ambulatory Care Reporting System), and the rate of hospital admissions for ambulatory care sensitive conditions (Discharge Abstract Database). Because the likelihood of using the emergency room and having a hospitalization is strongly tied to rurality, [13] we limited the evaluation of these measures to patients living in urban regions (Rurality Index of Ontario < 10). Continuity outcomes were the mean Usual Provider Care Index and the Practice Care Index, which represent the proportion of patients’ primary care visits made to their designated provider and within the practice of their designated provider, respectively. Chronic disease management outcomes captured whether or not patients received care (medication and tests) according to recommended guidelines. Tests were obtained from the OHIP billings and medication from the Ontario Drug Benefit database which contains prescription information of individuals for whom medication is covered by the provincial insurance; those on social assistance and all those > 65 years. Additional details on these indicators are presented in Additional file 1.

### Analyses

We described the patient profile, provider characteristics and quality of care measures for participating (IDOCC) and non-participating (Non-IDOCC) physicians. We conducted hierarchical regressions with patients nested within providers for access (Poisson), continuity (linear), and chronic disease management (logistic) indicators. For each quality of care indicator, we used a stepped regression approach to assess differences across IDOCC’s participants and non-participants. We evaluated the relationship without adjustment (Model 1), accounted for patient factors (Model 2), and then added physician factors (Model 3) in the regression model. Model 2 allowed a “fairer” comparison between the groups, while Model 3 allowed us to understand the impact of provider factors on the differences in performance across groups.

## Results

Table 2 reports descriptive statistics for the two groups. Capitation-based practices make up 13% of all Champlain practices, but 37% of participating practices. Similarly, family physicians working under the capitation model (45% vs. 18%), in rural practices (19% vs. 10%), as well as female physicians (55% vs. 47%), non-solo practitioners (92% vs. 73%), and to a lesser extent Canadian trained physicians (88% vs. 83%) and physicians with larger mean panel sizes (1,239 (1,154–1324) vs. 1,109 (1,061-1,158)) were more represented among IDOCC participants. Patient profiles differed little across groups.

**Table 2:** Profile of the study population. Description of the practices, providers and patients measures in each group

Practice Profile n	Total eligible	IDOCC	Non-IDOCC
	302	52	290
Model (n [%]) FFS	264 (87.4)	33 (63.5)	253 (87.2)
CAP non FHT	25 (8.3)	13 (25.0)	24 (8.3)
CAP and FHT	13 (4.3)	6 (11.5)	13 (4.5)
Provider Profile n	1,028	144	884
Model (n [%])			
FFS	808 (78.6)	79 (54.9)	729 (82.5)
CAP non Interp	115 (11.2)	37 (25.7)	78 (8.8)
CAP Interp	105 (10.2)	28 (19.4)	77 (8.7)
Foreign trained (n [%])	170 (16.6)	18 (12.5)	152 (17.2)
Male (n [%])	536 (52.1)	65 (45.1)	471 (53.3)
Group size (median)	11	11	11
1 (n [%])	251 (24.4)	11 (7.6)	240 (27.1)
2–10 (n [%])	248 (24.1)	51 (35.4)	197 (22.3)
11+ (n [%])	529 (51.5)	82 (56.9)	447 (50.6)
Rural (RIO > 40)	117 (11.4)	28 (19.4)	89 (10.1)
Panel size (mean, 95% CI)	1128 (1085–1172)	1239 (1154–1324)	1109 (1061–1158)
Age (mean, 95% CI)	48.8 (47.1–50.4)	49.5 (48.7–50.3)	49.4 (48.7–50.1)
Patient Profile n	1,103,491	174,769	928,722
Male (%)	515311 (46.7)	76,637 (43.9)	438,674 (47.2)
Age (mean, 95% CI)	40.2 (40.1–40.3)	40.3 (40.3–40.3)	40.3 (40.2–40.3)
RUBs (%)			
0	25231 (2.3)	4925 (2.8)	20306 (2.2)
1	73539 (6.7)	11148 (6.4)	62391 (6.7)
2	230717 (20.9)	36342 (20.8)	194375 (20.9)
3	570556 (51.7)	89735 (51.3)	480821 (51.8)
4	151202 (13.7)	24385 (14.0)	126817 (13.7)
5	52246 (4.7)	8234 (4.7)	44012 (4.7)
Rurality			
Urban (RIO < 10)	778850 (70.6)	116954 (66.9)	661896 (71.3)
Suburban (RIO 10–39)	170227 (15.4)	29745 (17.0)	140482 (15.1)
Rural (RIO ≥ 40)	154414 (14.0)	28070 (16.1)	126344 (13.6)
Income quintile (%)			
1	170805 (15.6)	21712 (12.5)	149093 (16.2)
2	205534 (18.8)	30460 (17.5)	175074 (19)
3	214141 (19.6)	34259 (19.7)	179882 (19.5)
4	253077 (23.1)	41179 (23.7)	211898 (23.0)
5	251673 (23.0)	46286 (26.6)	205387 (22.3)
Immigrant	115926 (10.5)	13566 (7.8)	102360 (11.0)

Profile of practices, physicians and patients meeting the IDOCC eligibility criteria across participation group

Note: In most (42) practices, not all physicians were involved in IDOCC. These are therefore represented in the IDOCC and non-IDOCC columns

95% CI = 95% confidence interval

IDOCC = Improved Delivery of Cardiovascular Care; FFS = Fee For Service; FHT = Family Health Team; CAP = Capitation; Interp = Interprofessional (note only CAP practices could be interprofessional); RIO = Rurality Index for Ontario; RUBs = Resource Utilization Bands, a measure of patient complexity

Note that because most practices (40) in which at least one physician participated in IDOCC also had physicians not participating in IDOCC, these practices are represented in the two columns, and total number of practices is not the sum of the two columns

Table 3 reports the estimated ratios measuring differences in quality of care outcomes between participating and non-participating physicians using the three modelling approaches described above. Additional file 2 provides the complete regression results model for the 14 outcomes considered. The direction of effect for all quality indicators was in favour of IDOCC physicians providing better care at baseline. After accounting for patient factors, the difference was statistically significant for two indicators within each dimension: 1) Cancer screening: cervical (Odds Ratio [95% confidence interval] (OR): 1.32 [1.08–1.61] and breast (OR: 1.32 [1.19–1.46]); 2) Access: emergency room visits (Rate Ratio (RR): 0.93 [0.88–0.97] and hospitalizations for ambulatory care sensitive conditions (RR:0.87 [0.77–0.99]; 3) Chronic disease management: eye exam (OR: 1.15 [1.06–1.25] and a glycated haemoglobin test (OR: 1.25 [1.06–1.49]); and 4) Continuity: provider (OR: 1.06 [1.03–1.09]) and practice (OR: 1.06 [1.04–1.08]) level.

**Table 3:** Association between IDOCC participation and quality of care indicators. Results of regression analyses showing the odds ratio of having a manoeuvre performed in patients of IDOCC participants compared to those of non-IDOCC participants for Cancer screening, Access, Chronic disease management and continuity measures

	N	Overall	Model 1 (Unadjusted)	*P* value	Model 2 (+ Patient)	*P* value	Model 3 (+ Patient/Physician)	*P* value
Cancer screening (%)								
Cervical	356820	66	**1.28 (1.13–1.45)**	<0.001	**1.32 (1.08–1.61)**	0.01	**1.16 (1.01–1.34)**	0.04
Colorectal	213015	41	**1.28 (1.11–1.47)**	<0.001	1.13 (0.94–1.36)	0.20	1.04 (0.87–1.25)	0.64
Breast	119162	68	**1.34 (1.21–1.49)**	<0.001	**1.32 (1.19–1.46)**	<0.001	**1.15 (1.05–1.26)**	< 0.01
Access (mean over 2 years)								
All ER visits (/100 indiv)	770491	54.4	0.96 (0.89–1.04)	0.32	**0.93 (0.88–0.97)**	<0.01	**0.93 (0.89–0.98)**	< 0.01
Low urgency ER visits (/100 indiv)	770491	19.8	0.93 (0.82–1.07)	0.31	0.94 (0.87–1.02)	0.15	0.92 (0.84–1.01)	0.07
Hospitalizations (/1000 indiv)	770491	5.1	0.90 (0.74–1.09)	0.28	**0.87 (0.77–0.99)**	0.03	**0.85 (0.75–0.97)**	0.01
Chronic Disease Management (Diabetes, %)								
Eye Exam test	63478	71	**1.17 (1.08–1.26)**	<0.001	**1.15 (1.06–1.25)**	<0.01	1.07 (0.98–1.17)	0.11
HgA1c test	63472	33	**1.20 (1.02–1.41)**	0.02	**1.25 (1.06–1.49)**	0.01	1.14 (0.96–1.35)	0.13
Lipid Tests	63472	58	1.11 (0.94–1.30)	0.21	1.10 (0.93–1.29)	0.27	1.06 (0.90–1.25)	0.46
ACE inhib or ARB agent	32874	71	1.08 (0.98–1.20)	0.11	1.07 (0.97–1.19)	0.17	1.02 (0.92–1.13)	0.72
Lipid Lowering agent	32874	66	1.05 (0.94–1.18)	0.37	1.05 (0.93–1.18)	0.46	1.01 (0.90–1.14)	0.82
Metformin	1815	86	1.24 (0.83–1.86)	0.29	1.16 (0.78–1.72)	0.47	1.16 (0.77–1.76)	0.47
Continuity (%)								
Usual Provider Care Index	808911	69	**1.07 (1.04–1.10)**	<0.001	**1.06 (1.03–1.09)**	<0.001	**1.06 (1.03–1.09)**	< 0.001
Practice Care Index	808911	77	**1.07 (1.04–1.09)**	<0.001	**1.06 (1.04–1.08)**	<0.001	**1.04 (1.02–1.06)**	< 0.001

Shows the odds ratio (cancer screening, chronic disease management), rate ratio (access measures and comprehensiveness) and risk difference (continuity) and 95% confidence intervals in brackets for IDOCC participants compared to non-IDOCC participants

Statistically significant differences in favour of IDOCC participants are bolded. There were no statistically significant differences in the other direction

ER = Emergency Room

Low urgency ER visits = ER visits triaged to urgency level 4 or 5 (out of a five point scale, with 5 being the least urgent)

Hospitalizations = Admissions for conditions deemed “ambulatory care sensitive”

Emergency room visits are reported per 100 and 1000 individuals, respectively

The continuity “Care Indices” represent the proportion of primary care visits made to the physician to whom they are assigned (Usual Provider) or the practice in which that physician works (Practice)

ACE inhib = Angiotensin Converting Enzyme Inhibitor; ARB = Angiotensin Receptor Blocker

Medications are only evaluated in patients > 65 years and those receiving social assistance

Adjusting for physician factors had no effect on access (RR for emergency room visits: 0.93 [0.88–0.98] and hospitalizations for ambulatory care sensitive conditions: 0.85 [0.75–0.97]) or continuity (OR for: 1.06 [1.03–1.09] and practice: 1.04 [1.02–1.06]) measures, but attenuated the effect observed in cancer screening **(**OR for: 1.16 [1.01–1.34] and breast: 1.15 [1.05–1.26]**)** and rendered the chronic disease management indicators non-significant. In these analyses, all included physician factors had a statistically significant association with at least one quality of care indicator (Additional file 2). Female physicians were associated with better quality of care across all but one indicator. Physicians who were younger, received their training in Canada, and belonged to group practices were usually, but not consistently, associated with better quality of care (See Additional file 2).

## Discussion

This study is the first to demonstrate that primary care physicians who agree to participate in a voluntary QI research initiative have a better performance quality level at baseline compared to those who do not. Accounting for patient characteristics, all 14 Quality of Primary Care indicators covering the four dimensions studied favoured the IDOCC group, with eight reaching statistical significance.

The observed differences in family physician profiles across the two groups were largely consistent with previous publications. IDOCC participants were more likely to be female, [3] and work in rural, [4] non-solo, [4, 5] capitation practices. [14] They had a larger clinical load [5], were less likely to be foreign trained, [1] but did not differ in age, [3, 4] or the profile of their patients.

Some of these characteristics had previously been associated with better quality of care. Female family physicians are reported to provide better chronic disease prevention and management. [15–18] A study conducted by members of our team demonstrated lower emergency room visits and hospitalization amongst patients receiving their care from female family physicians. [19] Similarly, there is some evidence of better outcomes from larger practices and practices whose remuneration is capitation-based. [20–22] However, while including all measured physician characteristics attenuated some of the observed differences, suggesting that differences in physician profile likely mediate some of the disparities in performance level across the groups; it did not completely eliminate these differences. The inclusion of physician characteristics had little impact on the measures of continuity, and clinical outcome measures such as the risk of emergency room visits and hospitalizations. These results suggest that other unmeasured provider- or practice-level attributes may affect the likelihood of agreeing to participate in a quality improvement program and the quality of care delivered.

Studies have suggested that practices’ attributes influence their providers’ likelihood of participating in QI initiatives. Practices are more likely to participate in QI initiatives when they have less hierarchical leadership structures, provide supportive or less stressful working environments, encourage innovation, value collaboration, or have a quality champion. [23–28] A recent review describing the barriers that limit physicians’ participation in innovations suggested that, in addition to the well-documented issues of competing priorities and limited resources, physicians who do not engage in innovations may be unwilling to expand efforts towards a yet unproven approach. [29]

Voluntary QI research studies face a challenge due to a ceiling effect, as the physicians most inclined to participate often have the least room to improve their delivery of care. Consequently, QI programs rarely achieve meaningful impact. [30] A systematic review found that QI initiatives aimed at improving diabetic control were most effective in practices that initially demonstrated poor performance. [31]

Recruitment strategies need to reach physicians and practices most likely to benefit from QI initiatives. However, more aggressive recruitment strategies or mandatory participation alone are unlikely to be sufficient, as achieving improvement in quality often requires investment in time and commitment by practice members to result in change. [32] Physicians disinclined to participate may be less invested in the initiative and achieve little improvement despite having access to the knowledge and resources provided by the QI program. The notion that a systematic approach to quality improvement is a fundamental part of practice remains relatively new, and it may take some years before it becomes part of most physicians’ professional identity. [5]

### Study limitations

We relied on a pragmatic trial implemented across a geographically diverse region in which over 1,000 family physicians working in different primary care models deliver care to approximately 1.2 million individuals. The study achieved participation rates comparable to other QI research programs. We used health administrative data to conduct a population-based evaluation capturing a broad range of quality of care outcomes. Because diabetes has high prevalence, and the guidelines for its management are well established and easily measured using health administrative data, it is commonly used to assess the quality of chronic conditions in population based studies. A broader set of indicators related to chronic conditions would have provided a more in depth assessment. We were not able to include CHCs in our comparisons and these made up nearly one quarter of our participants. The quality of care measures we assessed and the patient and provider profiles we analyzed were limited to those available in health administrative data. Factors that contribute to a physician’s decision to participate or not in a QI initiative may be related to the healthcare system context in which they operate. The results of this may not be entirely generalizable to other contexts.

## Conclusions

The participants who voluntarily agreed to participate in the IDOCC study differed in several ways from those who refused. The family physicians who agree to participate have a better performance quality level at baseline compared to those who do not. While characteristics of participating physicians can explain some of these differences, other underlying physician or practice attributes are also likely to be influencing both interest in QI initiatives and existing quality levels. QI initiatives are not adequately reaching their target population. Our results may help inform policymakers to develop effective recruitment and engagement strategies for similar programs, with the hope of enhancing their efficiency and cost effectiveness.

It is important to understand the differences between the participating and non-participating physicians in order to evaluate the generalizability and relevance of the conclusions from studies that rely on voluntary participation. Comparisons of the groups are typically very limited because very little information is available from those who choose not to participate. In this particular circumstance where we were able to compare participants and non-participants much more extensively than usual, we found important differences demonstrating that the physicians that need the help most were the least likely to participate. Further research is warranted to determine how widespread is the participation bias caused by the voluntary nature of research participation.

## Additional files

Additional file 1: IDOCC Rep: Performance indicator. Operational definition of performance indicators for Cancer screening, Access, Chronic disease management and continuity. (DOCX 15 kb)

Additional file 2: IDOCC Rep: Details of regressions. Results of regressions models for Cancer screening, Access, Chronic disease management and continuity indicators. (DOCX 76 kb)

## Data Availability

The datasets used and/or analysed during the current study are available from the corresponding author on reasonable request.

## Abbreviations

ICES: Institute for Clinical Evaluative Sciences
IDOCC: Improved Delivery of Cardiovascular Care
IPDB: ICES Physician Database
LHIN: Local Health Integration Network
OHIP: Ontario Health Insurance Program
QI: Quality Improvement

## References

[1] Wall TC, Mian MA, Ray MN, Casebeer L, Collins BC, Kiefe CI (2005). Improving physician performance through internet-based interventions: who will participate?. J Med Internet Res.

[2] van Heuvelen MJG, Hochstenbach JBM, Brouwer WH, de Greef MHG, Zijlstra GAR, van Jaarsveld E (2005). Differences between participants and non-participants in an RCT on physical activity and psychological interventions for older persons. Aging Clin Exp Res.

[3] Borgiel AE, Williams JI, Davis DA, Dunn EV, Hobbs N, Hutchison B (1999). Evaluating the effectiveness of 2 educational interventions in family practice. CMAJ.

[4] Shelton BJ, Wofford JL, Gosselink CA, McClatchey MW, Brekke K, Conry C (2002). Recruitment and retention of physicians for primary care research. J Community Health.

[5] Audet AM, Doty MM, Shamasdin J, Schoenbaum SC (2005). Measure, learn, and improve: physicians' involvement in quality improvement. Health Aff (Millwood ).

[6] Dahrouge S, Hogg W, Younger J, Muggah E, Russell G, Glazier RH (2016). Primary care physician panel size and quality of care: a population-based study in Ontario, Canada. Ann Fam Med.

[7] Jaakkimainen L, Klein-Geltink JE, Guttmann A, Barnsley J, Zagorski BM, Kopp A *et al*.: Indicators of primary care based on administrative data. In In Primary Care in Ontario. Institute for Clinical Evaluative Sciences; 2006:227.

[8] Liddy C, Hogg W, Russell G, Wells G, Armstrong C, Akbari A *et al*.: The improved delivery of cardiovascular care (IDOCC) through outreach facilitation: study protocol and implementation details of a cluster randomized controlled trial in primary care. BMC Implementation Science 2011, in print. 10.1186/1748-5908-6-110 PMC3197547 21952084

[9] Liddy C: A real-world stepped wedge cluster randomized trial of practice facilitation to improve cardiovascular care. Implement Sci 10*:*150*,* 2015 2015, 150. 10.1186/s13012-015-0341-y PMC4625868 26510577

[10] Dillman DA (1978). Mail and telephone surveys: the total design method.

[11] Reid R, Bogdanovic B, Roos NP, Black C, MacWilliam L, Menec V. Do some physician groups see sicker patients than others? Implications for primary care policy in Manitoba. 2001. Manitoba Centre for Health Policy and Evaluation: University of Manitoba.

[12] Wranik DW, Durier-Copp M (2010). Physician remuneration methods for family physicians in Canada: expected outcomes and lessons learned. Health Care Anal.

[13] Hossain Md. Monir, Laditka James N. (2011). The influence of rurality on the volume of non-urgent emergency department visits. Spatial and Spatio-temporal Epidemiology.

[14] Borgiel AE, Dunn EV, Lamont CT, MacDonald PJ, Evensen MK, Bass MJ (1989). Recruiting family physicians as participants in research. Fam Pract.

[15] Eisinger F, Pivot X, Coscas Y, Viguier J, Calazel-Benque A, Blay JY (2011). Impact of general practitioners’ sex and age on systematic recommendation for cancer screening. European Journal of Cancer Prevention.

[16] Ince-Cushman D, Correa JA, Shuldiner J, Segouin J (2013). Association of primary care physician sex with cervical cancer and mammography screening. Can Fam Physician.

[17] Lurie N, Slater J, McGovern P, Ekstrum J, Quam L, Margolis K (1993). Preventive care for women--does the sex of the physician matter?. N Engl J Med.

[18] Baumhakel M, Muller U, Bohm M (2009). Influence of gender of physicians and patients on guideline-recommended treatment of chronic heart failure in a cross-sectional study. Eur J Heart Fail.

[19] Dahrouge S, Seale E, Hogg W, Russell G, Younger J, Muggah E et al. (Eds):a comprehensive assessment of family physician gender and quality of care: a cross-sectional analysis in Ontario, Canada. In Med Care 2016. 10.1097/MLR.0000000000000480 26765146

[20] Beaulieu MD, Haggerty J, Tousignant P, Barnsley J, Hogg W, Geneau R (2013). Characteristics of primary care practices associated with high quality of care. Can Med Assoc J.

[21] Kiran T, Kopp A, Moineddin R, Glazier RH (2015). Longitudinal evaluation of physician payment reform and team-based care for chronic disease management and prevention. CMAJ.

[22] Nodora JN, Martz WD, Ashbeck EL, Jacobs ET, Thompson PA, Martinez ME (2011). Primary care physician compliance with colorectal cancer screening guidelines. Cancer Causes Control.

[23] Elovainio M, Steen N, Presseau J, Francis J, Hrisos S, Hawthorne G (2013). Is organizational justice associated with clinical performance in the care for patients with diabetes in primary care? Evidence from the improving quality of care in diabetes study. Fam Pract.

[24] Hung DY, Glasgow RE, Dickinson LM, Froshaug DB, Fernald DH, Balasubramanian BA (2008). The chronic care model and relationships to patient health status and health-related quality of life. Am J Prev Med.

[25] Luxford K., Safran D. G., Delbanco T. (2011). Promoting patient-centered care: a qualitative study of facilitators and barriers in healthcare organizations with a reputation for improving the patient experience. International Journal for Quality in Health Care.

[26] Quinn MA, Wilcox A, Orav EJ, Bates DW, Simon SR (2009). The relationship between perceived practice quality and quality improvement activities and physician practice dissatisfaction, professional isolation, and work-life stress. Med Care.

[27] Rushmer R, Kelly D, Lough M, Wilkinson JE, Davies HT (2004). Introducing the learning practice--I. the characteristics of learning organizations in primary care. J Eval Clin Pract.

[28] Siriwardena AN (2009). Engaging clinicians in quality improvement initiatives: art or science?. Qual Prim Care.

[29] Holmboe ES, Cassel CK (2007). The role of physicians and certification boards to improve quality. Am J Med Qual.

[30] Schouten LM, Hulscher ME, van Everdingen JJ, Huijsman R, Grol RP (2008). Evidence for the impact of quality improvement collaboratives: systematic review. [review] [15 refs]. BMJ.

[31] Tricco AC, Ivers NM, Grimshaw JM, Moher D, Turner L, Galipeau J (2012). Effectiveness of quality improvement strategies on the management of diabetes: a systematic review and meta-analysis. [review]. Lancet.

[32] Hogg W, Baskerville N, Nykiforuk C, Mallen D (2002). Improved preventive care in family practices with outreach facilitation: understanding success and failure. J Health Serv Res Policy.

