# Supplementary material for: High-performing physicians are more likely to participate in a research study: findings from a quality improvement study
Source: BMC Med Res Methodol. 2019 Aug 7;19:171. doi: 10.1186/s12874-019-0809-6 (PMC6685269; doi:10.1186/s12874-019-0809-6)
Supplement: Supplementary file 1 — IDOCC Rep: Performance indicator. Operational definition of performance indicators for Cancer screening, Access, Chronic disease management and continuity. (DOCX 15 kb) [file 12874_2019_809_MOESM1_ESM.docx]

**Appendix A: Performance Indicators**

| **Indicator** | **Definition** |
| --- | --- |
| **Cancer Screening** |  |
| Cervical cancer | Proportion of women ages 20-69 years who underwent a Papanicolaou test over 2 year observation period *[Source: OHIP, Cytobase]* |
| Breast cancer | Proportion of women ages 50-69 who underwent a mammography over 2 year observation period  *[Source: OHIP, OBSP]* |
| Colorectal cancer | Proportion of patients ages 50-74 who had a colorectal cancer screening investigation over 2 year observation period *[Source: OHIP]* |
| **Accessibility (urban residents only)** |  |
| Emergency department visits | Number (rate) of emergency room visits of any triage level, excluding planned visits *[Source: NACRS]* |
| Emergency room use for low triage | Number (rate) of emergency room visits for triage category 4 (semi-urgent) and category 5 (non-urgent) per patient, (over 2 year observation period), excluding planned visits *[Source: NACRS]* |
| Ambulatory care sensitive admissions | Number (rate) of admissions for ambulatory care sensitive conditions [asthma, congestive heart failure, chronic obstructive pulmonary disease (COPD), diabetes, or asthma/COPD] per 1000 patients (over 2 year observation period) *[Source: DAD]* |
| **Continuity** |  |
| Usual **Provider** of Care Index at the patient level (UPC-Patient) | The Usual Provider of Care Index represents the proportion of primary care visits to the provider to whom they are attributed (rostered or virtually rostered) relative to all primary care visits in the 2 year observation period. Because of the resulting clustering of results at 0%, 50% and 100% for individuals with less 3 visits, patients with < 3 visits are excluded *[Source: OHIP]* |
| **Practice** of Care Index at the patient level - (PCI-Patient) | The Usual Practice of Care Index represents the proportion of primary care visits to physicians in the practice of the provider to whom they are attributed (rostered or virtually rostered) relative to all primary care visits in the 2 year observation period. Because of the resulting clustering of results at 0%, 50% and 100% for individuals with less 3 visits, patients with < 3 visits are excluded *[Source: OHIP]* |
| **Chronic Disease Management** |  |
| **Diabetes** |  |
| Eye exam | Proportion of individuals age > 40 years at the start of the 2 year evaluation period with diabetes who have had an eye exam within the past 2 years *[Source: OHIP]* |
| Metformin | Proportion of individuals > 65 years of age diagnosed with diabetes between 1 and 2 years prior to study period who received metformin as a first hypoglycemic agent prescription *[Source: OHIP, ODB]* |
| Angiotensin receptor blockers (ARB) or Angiotensin converting enzyme (ACE) inhibitor | Proportion of individuals > 65 years of age with diabetes 1 year prior to the study period who received at least one prescription for ARB or ACE in past year *[Source: OHIP, ODB]* |
| Lipid-lowering agent | Proportion of individuals > 65 years of age with diabetes 1 year prior to the study period who received at least one prescription for statin in past year *[Source: OHIP, ODB]* |
| Lipids test | Proportion of individuals age > 40 years at the start of the 2 year evaluation period with diabetes who have had at least two lipids tests in the past 2 years *[Source: OHIP]* |
| HgA1c test | Proportion of individuals age > 40 years at the start of the 2 year evaluation period with diabetes who have had at least four HbA1c tests in the past 2 years *[Source: OHIP]* |
