# Supplementary material for: High-performing physicians are more likely to participate in a research study: findings from a quality improvement study
Source: BMC Med Res Methodol. 2019 Aug 7;19:171. doi: 10.1186/s12874-019-0809-6 (PMC6685269; doi:10.1186/s12874-019-0809-6)
Supplement: Supplementary file 2 — IDOCC Rep: Details of regressions. Results of regressions models for Cancer screening, Access, Chronic disease management and continuity indicators. (DOCX 76 kb) [file 12874_2019_809_MOESM2_ESM.docx]

**Appendix B: Details of regressions**

**Cervical Screening**

|  | Adjusted Patient Characteristics | | | | Fully Adjusted (Patient and Physician) | | | |
| --- | --- | --- | --- | --- | --- | --- | --- | --- |
|  | Estimate | Standard Error | 95% Confidence Limits | P-value | Estimate | Standard Deviation | 95% Confidence Limits | P-value |
| Age Groups |  |  |  |  |  |  |  |  |
| 20-49 | Referent |  |  |  | Referent |  |  |  |
| 50-64 | -0.38 | 0.01 | -0.41- -0.36 | <0.001 | -0.40 | 0.01 | -0.43- -0.38 | <0.001 |
| 65-69 | -0.99 | 0.02 | -1.04- -0.95 | <0.001 | -1.03 | 0.02 | -1.08- -0.99 | <0.001 |
| Income Quintile |  |  |  |  |  |  |  |  |
| 1 | -0.38 | 0.01 | -0.41- -0.36 | <0.001 | -0.40 | 0.01 | -0.43- -0.37 | <0.001 |
| 2 | -0.20 | 0.01 | -0.22- -0.18 | <0.001 | -0.21 | 0.01 | -0.24- -0.19 | <0.001 |
| 3 | -0.12 | 0.01 | -0.14- -0.10 | <0.001 | -0.13 | 0.01 | -0.15- -0.11 | <0.001 |
| 4 | -0.08 | 0.01 | -0.10- -0.06 | <0.001 | -0.09 | 0.01 | -0.11- -0.06 | <0.001 |
| 5 | Referent |  |  |  | Referent |  |  |  |
| Resource Utilization Band |  |  |  |  |  |  |  |  |
| 0 | Referent |  |  |  | Referent |  |  |  |
| 1 | 3.07 | 0.23 | 2.63- 3.52 | <0.001 | 2.98 | 0.17 | 2.64- 3.32 | <0.001 |
| 2 | 3.27 | 0.23 | 2.82- 3.71 | <0.001 | 3.18 | 0.17 | 2.84- 3.52 | <0.001 |
| 3 | 3.70 | 0.23 | 3.25- 4.15 | <0.001 | 3.64 | 0.17 | 3.29- 3.98 | <0.001 |
| 4 | 3.93 | 0.23 | 3.49- 4.38 | <0.001 | 3.88 | 0.18 | 3.54- 4.23 | <0.001 |
| 5 | 3.31 | 0.23 | 2.86- 3.76 | <0.001 | 3.23 | 0.18 | 2.88- 3.57 | <0.001 |
| Immigrant | -0.19 | 0.01 | -0.22- -0.17 | <0.001 | -0.20 | 0.01 | -0.23- -0.18 | <0.001 |
| Rurality |  |  |  |  |  |  |  |  |
| Urban | Referent |  |  |  | Referent |  |  |  |
| Suburban | -0.06 | 0.02 | -0.09- -0.03 | <0.001 | -0.07 | 0.02 | -0.10- -0.03 | <0.001 |
| Rural | -0.03 | 0.02 | -0.07- 0.01 | 0.11 | -0.03 | 0.02 | -0.07- 0.01 | 0.16 |
| Female Physician |  |  |  |  | 0.74 | 0.05 | 0.64- 0.84 | <0.001 |
| Physician Age |  |  |  |  | 0.01 | 0.003 | 0.001- 0.01 | 0.02 |
| Foreign Trained |  |  |  |  | -0.32 | 0.06 | -0.45- -0.20 | <0.001 |
| Practice Group Size |  |  |  |  |  |  |  |  |
| 1 |  |  |  |  | Referent |  |  |  |
| 2-10 |  |  |  |  | 0.34 | 0.09 | 0.17- 0.51 | <0.001 |
| 11+ |  |  |  |  | 0.41 | 0.07 | 0.27- 0.54 | <0.001 |
| Model |  |  |  |  |  |  |  |  |
| FFS |  |  |  |  | Referent |  |  |  |
| Capitation, non-FHT |  |  |  |  | -0.02 | 0.10 | -0.21- 0.17 | 0.85 |
| Capitation FHT |  |  |  |  | -0.18 | 0.09 | -0.36- -0.002 | 0.05 |
| Panel Size |  |  |  |  | 0.0002 | 0.00 | 0.0001- 0.0003 | <0.001 |

**Colorectal Screening**

|  | Adjusted Patient Characteristics | | | | Fully Adjusted (Patient and Physician) | | | |
| --- | --- | --- | --- | --- | --- | --- | --- | --- |
|  | Estimate | Standard Deviation | 95% Confidence Limits | P-value | Estimate | Standard Deviation | 95% Confidence Limits | P-value |
| Female | -0.01 | 0.01 | -0.04- 0.01 | 0.34 | -0.02 | 0.01 | -0.04- 0.01 | 0.22 |
| Age Groups |  |  |  |  |  |  |  |  |
| 50-64 | Referent |  |  |  | Referent |  |  |  |
| 65-74 | 0.25 | 0.01 | 0.23- 0.28 | <0.001 | 0.26 | 0.01 | 0.24- 0.29 | <.0001 |
| Income Quintile |  |  |  |  |  |  |  |  |
| 1 | -0.28 | 0.02 | -0.31- -0.25 | <0.001 | -0.29 | 0.02 | -0.32- -0.25 | <0.001 |
| 2 | -0.12 | 0.01 | -0.15- -0.10 | <0.001 | -0.13 | 0.01 | -0.15- -0.10 | <0.001 |
| 3 | -0.10 | 0.01 | -0.12- -0.07 | <0.001 | -0.10 | 0.01 | -0.13- -0.07 | <0.001 |
| 4 | -0.05 | 0.01 | -0.07- -0.03 | <0.001 | -0.05 | 0.01 | -0.08- -0.03 | <0.001 |
| 5 | Referent |  |  |  | Referent |  |  |  |
| Resource Utilization Band |  |  |  |  |  |  |  |  |
| 0 | Referent |  |  |  | Referent |  |  |  |
| 1 | 2.34 | 0.19 | 1.97- 2.72 | <0.001 | 2.28 | 0.17 | 1.94- 2.62 | <0.001 |
| 2 | 2.64 | 0.19 | 2.26- 3.01 | <0.001 | 2.58 | 0.17 | 2.24- 2.92 | <0.001 |
| 3 | 3.14 | 0.19 | 2.77- 3.52 | <0.001 | 3.10 | 0.17 | 2.76- 3.44 | <0.001 |
| 4 | 3.26 | 0.19 | 2.88- 3.64 | <0.001 | 3.22 | 0.17 | 2.88- 3.56 | <0.001 |
| 5 | 3.04 | 0.19 | 2.67- 3.42 | <0.001 | 3.00 | 0.17 | 2.66- 3.34 | <0.001 |
| Immigrant | -0.12 | 0.02 | -0.15- -0.08 | <0.001 | -0.12 | 0.02 | -0.16- -0.08 | <0.001 |
| Rurality |  |  |  |  |  |  |  |  |
| Urban | Referent |  |  |  | Referent |  |  |  |
| Suburban | -0.10 | 0.02 | -0.15- -0.06 | <0.001 | -0.10 | 0.02 | -0.15- -0.05 | <0.001 |
| Rural | -0.12 | 0.03 | -0.17- -0.06 | <0.001 | -0.11 | 0.03 | -0.16- -0.05 | <0.001 |
| Female Physician |  |  |  |  | 0.34 | 0.06 | 0.22- 0.47 | <0.001 |
| Physician Age |  |  |  |  | -0.01 | 0.003 | -0.01- -0.001 | 0.02 |
| Foreign Trained |  |  |  |  | -0.13 | 0.09 | -0.31- 0.05 | 0.15 |
| Practice Group Size |  |  |  |  |  |  |  |  |
| 1 |  |  |  |  | Referent |  |  |  |
| 2-10 |  |  |  |  | 0.61 | 0.12 | 0.38- 0.85 | <0.001 |
| 11+ |  |  |  |  | 0.7 | 0.09 | 0.52- 0.88 | <0.001 |
| Model |  |  |  |  |  |  |  |  |
| FFS |  |  |  |  | Referent |  |  |  |
| Capitation, non-FHT |  |  |  |  | -0.21 | 0.12 | -0.45- 0.03 | 0.08 |
| Capitation FHT |  |  |  |  | 0.02 | 0.10 | -0.18- 0.21 | 0.86 |
| Panel Size |  |  |  |  | 0.0001 | 0.0001 | 0.00- 0.0002 | 0.18 |

**Mammography**

|  | Adjusted Patient Characteristics | | | | Fully Adjusted (Patient and Physician) | | | |
| --- | --- | --- | --- | --- | --- | --- | --- | --- |
|  | Estimate | Standard Deviation | 95% Confidence Limits | P-value | Estimate | Standard Deviation | 95% Confidence Limits | P-value |
| Age Groups |  |  |  |  |  |  |  |  |
| 50-64 | Referent |  |  |  | Referent |  |  |  |
| 65-69 | 0.06 | 0.02 | 0.02- 0.09 | 0.0005 | 0.06 | 0.02 | 0.03- 0.09 | <0.001 |
| Income Quintile |  |  |  |  |  |  |  |  |
| 1 | -0.47 | 0.02 | -0.51- -0.42 | <0.001 | -0.47 | 0.02 | -0.51- -0.42 | <0.001 |
| 2 | -0.22 | 0.02 | -0.26- -0.18 | <0.001 | -0.22 | 0.02 | -0.26- -0.18 | <0.001 |
| 3 | -0.14 | 0.02 | -0.17- -0.10 | <0.001 | -0.14 | 0.02 | -0.18- -0.1 | <0.001 |
| 4 | -0.09 | 0.02 | -0.13- -0.06 | <0.001 | -0.09 | 0.02 | -0.13- -0.06 | <0.001 |
| 5 | Referent |  |  |  | Referent |  |  |  |
| Resource Utilization Band |  |  |  |  |  |  |  |  |
| 0 | Referent |  |  |  | Referent |  |  |  |
| 1 | 1.41 | 0.11 | 1.19- 1.63 | <0.001 | 1.42 | 0.11 | 1.2- 1.63 | <0.001 |
| 2 | 1.77 | 0.11 | 1.56- 1.98 | <0.001 | 1.78 | 0.10 | 1.58- 1.98 | <0.001 |
| 3 | 2.33 | 0.11 | 2.12- 2.54 | <0.001 | 2.36 | 0.10 | 2.16- 2.56 | <0.001 |
| 4 | 2.43 | 0.11 | 2.22- 2.64 | <0.001 | 2.46 | 0.10 | 2.26- 2.66 | <0.001 |
| 5 | 2.05 | 0.11 | 1.83- 2.27 | <0.001 | 2.07 | 0.11 | 1.86- 2.28 | <0.001 |
| Immigrant | -0.33 | 0.03 | -0.38- -0.28 | <0.001 | -0.33 | 0.03 | -0.38- -0.28 | <0.001 |
| Rurality |  |  |  |  |  |  |  |  |
| Urban | Referent |  |  |  | Referent |  |  |  |
| Suburban | -0.05 | 0.03 | -0.11- -0.004 | 0.03 | -0.05 | 0.03 | -0.1- 0.004 | 0.07 |
| Rural | -0.01 | 0.03 | -0.07- 0.04 | 0.66 | 0.02 | 0.03 | -0.04- 0.08 | 0.45 |
| Female Physician |  |  |  |  | 0.40 | 0.04 | 0.32- 0.47 | <0.001 |
| Physician Age |  |  |  |  | -0.001 | 0.002 | -0.005- 0.003 | 0.62 |
| Foreign Trained |  |  |  |  | -0.19 | 0.05 | -0.29- -0.09 | <0.001 |
| Practice Group Size |  |  |  |  |  |  |  |  |
| 1 |  |  |  |  | Referent |  |  |  |
| 2-10 |  |  |  |  | 0.24 | 0.06 | 0.11- 0.36 | <0.001 |
| 11+ |  |  |  |  | 0.29 | 0.06 | 0.18- 0.4 | <0.001 |
| Model |  |  |  |  |  |  |  |  |
| FFS |  |  |  |  | Referent |  |  |  |
| Capitation, non-FHT |  |  |  |  | 0.12 | 0.05 | 0.01- 0.22 | 0.03 |
| Capitation FHT |  |  |  |  | 0.25 | 0.05 | 0.14- 0.35 | <.0001 |
| Panel Size |  |  |  |  | 0.0001 | 0.00 | 0- 0.0001 | 0.10 |

**Emergency Department Visits- Urban Patients**

|  | Adjusted Patient Characteristics | | | | Fully Adjusted (Patient and Physician) | | | |
| --- | --- | --- | --- | --- | --- | --- | --- | --- |
|  | Estimate | Standard Deviation | 95% Confidence Limits | P-value | Estimate | Standard Deviation | 95% Confidence Limits | P-value |
| Female | -0.09 | 0.01 | -0.10- -0.08 | <0.001 | -0.08 | 0.01 | -0.1- -0.07 | <0.001 |
| Age Groups |  |  |  |  |  |  |  |  |
| <2 | Referent |  |  |  | Referent |  |  |  |
| 2-17 | 0.32 | 0.02 | 0.28- 0.35 | <0.001 | 0.31 | 0.02 | 0.27- 0.35 | <0.001 |
| 18-49 | -0.08 | 0.02 | -0.11- -0.04 | <0.001 | -0.08 | 0.02 | -0.12- -0.05 | <0.001 |
| 50-64 | -0.42 | 0.02 | -0.46- -0.39 | <0.001 | -0.43 | 0.02 | -0.47- -0.40 | <0.001 |
| 65-74 | -0.51 | 0.02 | -0.55- -0.48 | <0.001 | -0.52 | 0.02 | -0.56- -0.49 | <0.001 |
| 75+ | -0.36 | 0.02 | -0.39- -0.32 | <0.001 | -0.37 | 0.02 | -0.41- -0.33 | <0.001 |
| Income Quintile |  |  |  |  |  |  |  |  |
| 1 | 0.37 | 0.01 | 0.36- 0.39 | <0.001 | 0.37 | 0.01 | 0.35- 0.39 | <0.001 |
| 2 | 0.22 | 0.01 | 0.21- 0.24 | <0.001 | 0.22 | 0.01 | 0.21- 0.24 | <0.001 |
| 3 | 0.14 | 0.01 | 0.13- 0.15 | <0.001 | 0.14 | 0.01 | 0.12- 0.15 | <0.001 |
| 4 | 0.04 | 0.01 | 0.03- 0.06 | <0.001 | 0.04 | 0.01 | 0.03- 0.06 | <0.001 |
| 5 | Referent |  |  |  | Referent |  |  |  |
| Resource Utilization Band |  |  |  |  |  |  |  |  |
| ≤1 | Referent |  |  |  | Referent |  |  |  |
| 2 | 1.07 | 0.02 | 1.03- 1.10 | <0.001 | 1.06 | 0.02 | 1.03- 1.10 | <0.001 |
| 3 | 1.96 | 0.02 | 1.91- 2.00 | <0.001 | 1.96 | 0.02 | 1.91- 2.00 | <0.001 |
| 4 | 2.82 | 0.03 | 2.76- 2.87 | <0.001 | 2.82 | 0.03 | 2.77- 2.87 | <0.001 |
| 5 | 3.73 | 0.03 | 3.66- 3.79 | <0.001 | 3.72 | 0.03 | 3.66- 3.78 | <0.001 |
| Immigrant | -0.26 | 0.01 | -0.27- -0.24 | <0.001 | -0.26 | 0.01 | -0.27- -0.24 | <0.001 |
| Female Physician |  |  |  |  | -0.16 | 0.02 | -0.19- -0.12 | <0.001 |
| Physician Age |  |  |  |  | -0.0003 | 0.001 | -0.003- 0.002 | 0.81 |
| Foreign Trained |  |  |  |  | 0.02 | 0.03 | -0.04- 0.09 | 0.45 |
| Practice Group Size |  |  |  |  |  |  |  |  |
| 1 |  |  |  |  | Referent |  |  |  |
| 2-10 |  |  |  |  | -0.03 | 0.03 | -0.09- 0.03 | 0.26 |
| 11+ |  |  |  |  | -0.04 | 0.03 | -0.10- 0.02 | 0.17 |
| Model |  |  |  |  |  |  |  |  |
| FFS |  |  |  |  | Referent |  |  |  |
| Capitation, non-FHT |  |  |  |  | 0.22 | 0.04 | 0.15- 0.29 | <0.001 |
| Capitation FHT |  |  |  |  | -0.03 | 0.03 | -0.09- 0.02 | 0.22 |
| Panel Size |  |  |  |  | 0.00 | 0.00 | 0.00- 0.00 | 0.35 |

**Low Triage Emergency Department Visits- Urban Patients**

|  | Adjusted Patient Characteristics | | | | Fully Adjusted (Patient and Physician) | | | |
| --- | --- | --- | --- | --- | --- | --- | --- | --- |
|  | Estimate | Standard Deviation | 95% Confidence Limits | P-value | Estimate | Standard Deviation | 95% Confidence Limits | P-value |
| Female | -0.11 | 0.01 | -0.12- -0.10 | <0.001 | -0.11 | 0.01 | -0.12- -0.09 | <0.001 |
| Age Groups |  |  |  |  |  |  |  |  |
| <2 | Referent |  |  |  | Referent |  |  |  |
| 2-17 | 0.78 | 0.03 | 0.73- 0.83 | <0.001 | 0.79 | 0.03 | 0.74- 0.84 | <0.001 |
| 18-49 | 0.29 | 0.02 | 0.25- 0.34 | <0.001 | 0.30 | 0.02 | 0.25- 0.34 | <0.001 |
| 50-64 | -0.17 | 0.02 | -0.21- -0.12 | <0.001 | -0.17 | 0.02 | -0.21- -0.12 | <0.001 |
| 65-74 | -0.36 | 0.02 | -0.41- -0.32 | <0.001 | -0.37 | 0.03 | -0.42- -0.32 | <0.001 |
| 75+ | -0.37 | 0.03 | -0.42- -0.32 | <0.001 | -0.38 | 0.03 | -0.43- -0.33 | <0.001 |
| Income Quintile |  |  |  |  |  |  |  |  |
| 1 | 0.34 | 0.01 | 0.32- 0.36 | <0.001 | 0.35 | 0.01 | 0.33- 0.37 | <0.001 |
| 2 | 0.23 | 0.01 | 0.21- 0.25 | <0.001 | 0.23 | 0.01 | 0.21- 0.25 | <0.001 |
| 3 | 0.17 | 0.01 | 0.15- 0.18 | <0.001 | 0.17 | 0.01 | 0.15- 0.18 | <0.001 |
| 4 | 0.03 | 0.01 | 0.01- 0.04 | <0.001 | 0.03 | 0.01 | 0.01- 0.04 | <0.001 |
| 5 | Referent |  |  |  | Referent |  |  |  |
| Resource Utilization Band |  |  |  |  |  |  |  |  |
| ≤1 | Referent |  |  |  | Referent |  |  |  |
| 2 | 0.86 | 0.02 | 0.82- 0.91 | <0.001 | 0.87 | 0.02 | 0.83- 0.91 | <0.001 |
| 3 | 1.56 | 0.03 | 1.5- 1.61 | <0.001 | 1.57 | 0.03 | 1.52- 1.62 | <0.001 |
| 4 | 2.18 | 0.03 | 2.12- 2.25 | <0.001 | 2.21 | 0.03 | 2.15- 2.27 | <0.001 |
| 5 | 2.81 | 0.04 | 2.73- 2.90 | <0.001 | 2.84 | 0.04 | 2.76- 2.91 | <0.001 |
| Immigrant | -0.30 | 0.01 | -0.32- -0.28 | <0.001 | -0.30 | 0.01 | -0.32- -0.28 | <0.001 |
| Female Physician |  |  |  |  | -0.18 | 0.03 | -0.25- -0.11 | <0.001 |
| Physician Age |  |  |  |  | -0.003 | 0.002 | -0.01- 0 | 0.10 |
| Foreign Trained |  |  |  |  | -0.01 | 0.05 | -0.1- 0.08 | 0.79 |
| Practice Group Size |  |  |  |  |  |  |  |  |
| 1 |  |  |  |  | Referent |  |  |  |
| 2-10 |  |  |  |  | -0.08 | 0.05 | -0.19- 0.02 | 0.12 |
| 11+ |  |  |  |  | -0.13 | 0.05 | -0.23- -0.03 | 0.01 |
| Model |  |  |  |  |  |  |  |  |
| FFS |  |  |  |  | Referent |  |  |  |
| Capitation, non-FHT |  |  |  |  | 0.43 | 0.07 | 0.3- 0.56 | <0.001 |
| Capitation FHT |  |  |  |  | -0.03 | 0.05 | -0.12- 0.07 | 0.61 |
| Panel Size |  |  |  |  | 0.00 | 0.00 | 0.00- 0.0001 | 0.04 |

**Ambulatory Care Sensitive Hospital Admissions in Urban Patients**

|  | Adjusted Patient Characteristics | | | | Fully Adjusted (Patient and Physician) | | | |
| --- | --- | --- | --- | --- | --- | --- | --- | --- |
|  | Estimate | Standard Deviation | 95% Confidence Limits | P-value | Estimate | Standard Deviation | 95% Confidence Limits | P-value |
| Female | -0.10 | 0.04 | -0.18- -0.03 | 0.01 | -0.08 | 0.04 | -0.15- 0.002 | 0.06 |
| Age Groups |  |  |  |  |  |  |  |  |
| <2 | Referent |  |  |  | Referent |  |  |  |
| 2-17 | 1.16 | 0.27 | 0.64- 1.69 | <0.001 | 1.17 | 0.27 | 0.64- 1.69 | <0.001 |
| 18-49 | 0.06 | 0.26 | -0.44- 0.57 | 0.80 | 0.05 | 0.26 | -0.45- 0.56 | 0.85 |
| 50-64 | 0.88 | 0.26 | 0.38- 1.38 | <0.001 | 0.86 | 0.26 | 0.35- 1.36 | <0.001 |
| 65-74 | 1.45 | 0.26 | 0.94- 1.96 | <0.001 | 1.43 | 0.26 | 0.91- 1.94 | <0.001 |
| 75+ | 1.89 | 0.26 | 1.39- 2.39 | <0.001 | 1.86 | 0.26 | 1.35- 2.37 | <0.001 |
| Income Quintile |  |  |  |  |  |  |  |  |
| 1 | 0.83 | 0.06 | 0.71- 0.95 | <0.001 | 0.83 | 0.06 | 0.71- 0.96 | <0.001 |
| 2 | 0.48 | 0.06 | 0.36- 0.60 | <0.001 | 0.49 | 0.06 | 0.37- 0.61 | <0.001 |
| 3 | 0.30 | 0.06 | 0.18- 0.43 | <0.001 | 0.30 | 0.07 | 0.17- 0.42 | <0.001 |
| 4 | 0.16 | 0.06 | 0.04- 0.29 | 0.01 | 0.16 | 0.06 | 0.04- 0.29 | 0.01 |
| 5 | Referent |  |  |  | Referent |  |  |  |
| Resource Utilization Band |  |  |  |  |  |  |  |  |
| ≤2 | Referent |  |  |  | Referent |  |  |  |
| 3 | 2.15 | 0.19 | 1.77- 2.53 | <0.001 | 2.16 | 0.20 | 1.78- 2.55 | <0.001 |
| 4 | 3.85 | 0.19 | 3.48- 4.22 | <0.001 | 3.87 | 0.19 | 3.49- 4.24 | <0.001 |
| 5 | 5.37 | 0.19 | 4.99- 5.75 | <0.001 | 5.38 | 0.19 | 5.00- 5.76 | <0.001 |
| Immigrant | -0.35 | 0.09 | -0.53- -0.18 | <0.001 | -0.35 | 0.09 | -0.53- -0.18 | <0.001 |
| Female Physician |  |  |  |  | -0.18 | 0.05 | -0.28- -0.09 | <0.01 |
| Physician Age |  |  |  |  | -0.002 | 0.002 | -0.01- 0.003 | 0.38 |
| Foreign Trained |  |  |  |  | 0.02 | 0.07 | -0.11- 0.15 | 0.72 |
| Practice Group Size |  |  |  |  |  |  |  |  |
| 1 |  |  |  |  | Referent |  |  |  |
| 2-10 |  |  |  |  | -0.08 | 0.08 | -0.24- 0.09 | 0.36 |
| 11+ |  |  |  |  | -0.01 | 0.07 | -0.14- 0.12 | 0.88 |
| Model |  |  |  |  |  |  |  |  |
| FFS |  |  |  |  | Referent |  |  |  |
| Capitation, non-FHT |  |  |  |  | 0.32 | 0.09 | 0.14- 0.49 | 0.00 |
| Capitation FHT |  |  |  |  | 0.01 | 0.07 | -0.13- 0.15 | 0.92 |
| Panel Size |  |  |  |  | -0.0001 | 0.00 | -0.0001- 0.00 | 0.18 |

**Continuity of Care with Physician**

|  | Adjusted Patient Characteristics | | | | Fully Adjusted (Patient and Physician) | | | |
| --- | --- | --- | --- | --- | --- | --- | --- | --- |
|  | Estimate | Standard Deviation | 95% Confidence Limits | P-value | Estimate | Standard Deviation | 95% Confidence Limits | P-value |
| Female | -0.01 | 0.001 | -0.01- -0.01 | <0.001 | -0.01 | 0.001 | -0.01- -0.01 | <0.001 |
| Age Groups |  |  |  |  |  |  |  |  |
| <2 | Referent |  |  |  | Referent |  |  |  |
| 2-17 | -0.19 | 0.004 | -0.19- -0.18 | <0.001 | -0.19 | 0.004 | -0.19- -0.18 | <0.001 |
| 18-49 | -0.14 | 0.004 | -0.15- -0.14 | <0.001 | -0.14 | 0.004 | -0.15- -0.14 | <0.001 |
| 50-64 | -0.04 | 0.004 | -0.05- -0.04 | <0.001 | -0.04 | 0.004 | -0.05- -0.04 | <0.001 |
| 65-74 | -0.01 | 0.004 | -0.02- 0.0001 | 0.05 | -0.01 | 0.004 | -0.02- 0.00 | 0.05 |
| 75+ | -0.02 | 0.005 | -0.02- -0.01 | <0.001 | -0.02 | 0.005 | -0.02- -0.01 | <0.001 |
| Income Quintile |  |  |  |  |  |  |  |  |
| 1 | -0.01 | 0.002 | -0.01- -0.003 | <0.001 | -0.01 | 0.002 | -0.01- -0.003 | <0.001 |
| 2 | -0.001 | 0.001 | -0.003- 0.002 | 0.54 | -0.0007 | 0.001 | -0.003- 0.002 | 0.56 |
| 3 | -0.001 | 0.001 | -0.003- 0.001 | 0.43 | -0.0009 | 0.001 | -0.003- 0.001 | 0.44 |
| 4 | -0.001 | 0.001 | -0.003- 0.001 | 0.49 | -0.0007 | 0.001 | -0.003- 0.001 | 0.50 |
| 5 | Referent |  |  |  | Referent |  |  |  |
| Resource Utilization Band |  |  |  |  |  |  |  |  |
| 0 | Referent |  |  |  | Referent |  |  |  |
| 1 | -0.14 | 0.06 | -0.26- -0.03 | 0.01 | -0.14 | 0.058 | -0.26- -0.03 | 0.01 |
| 2 | -0.14 | 0.06 | -0.25- -0.02 | 0.02 | -0.14 | 0.059 | -0.25- -0.02 | 0.02 |
| 3 | -0.16 | 0.06 | -0.27- -0.04 | <0.01 | -0.16 | 0.059 | -0.27- -0.04 | 0.01 |
| 4 | -0.18 | 0.06 | -0.3- -0.07 | <0.01 | -0.18 | 0.058 | -0.3- -0.07 | <0.01 |
| 5 | -0.21 | 0.06 | -0.33- -0.10 | <0.001 | -0.21 | 0.059 | -0.33- -0.10 | <0.001 |
| Immigrant | 0.02 | 0.002 | 0.02- 0.03 | <0.001 | 0.02 | 0.002 | 0.02- 0.03 | <0.001 |
| Rurality |  |  |  |  |  |  |  |  |
| Urban | Referent |  |  |  | Referent |  |  |  |
| Suburban | 0.05 | 0.003 | 0.04- 0.06 | <0.001 | 0.05 | 0.003 | 0.04- 0.06 | <0.001 |
| Rural | 0.06 | 0.004 | 0.05- 0.07 | <0.001 | 0.06 | 0.004 | 0.05- 0.07 | <0.001 |
| Female Physician |  |  |  |  | 0.01 | 0.01 | -0.01- 0.03 | 0.01 |
| Physician Age |  |  |  |  | 0.005 | 0.001 | 0.004- 0.01 | <0.001 |
| Foreign Trained |  |  |  |  | -0.06 | 0.013 | -0.08- -0.03 | <0.001 |
| Practice Group Size |  |  |  |  |  |  |  |  |
| 1 |  |  |  |  | Referent |  |  |  |
| 2-10 |  |  |  |  | 0.03 | 0.016 | -0.002- 0.06 | 0.07 |
| 11+ |  |  |  |  | 0.01 | 0.014 | -0.01- 0.04 | 0.32 |
| Model |  |  |  |  |  |  |  |  |
| FFS |  |  |  |  | Referent |  |  |  |
| Capitation, non-FHT |  |  |  |  | -0.02 | 0.017 | -0.05- 0.02 | 0.31 |
| Capitation FHT |  |  |  |  | -0.17 | 0.018 | -0.20- -0.13 | <0.001 |
| Panel Size |  |  |  |  | 0.0001 | 0.00 | 0.0001- 0.0001 | <0.001 |

**Continuity of Care at the Practice Level**

|  | Adjusted Patient Characteristics | | | | Fully Adjusted (Patient and Physician) | | | |
| --- | --- | --- | --- | --- | --- | --- | --- | --- |
|  | Estimate | Standard Deviation | 95% Confidence Limits | P-value | Estimate | Standard Deviation | 95% Confidence Limits | P-value |
| Female | -0.01 | 0.001 | -0.01- -0.01 | <0.001 | -0.01 | 0.001 | -0.01- -0.01 | <.0001 |
| Age Groups |  |  |  |  |  |  |  |  |
| <2 | Referent |  |  |  | Referent |  |  |  |
| 2-17 | -0.15 | 0.004 | -0.16- -0.14 | <0.001 | -0.15 | 0.004 | -0.16- -0.14 | <.0001 |
| 18-49 | -0.14 | 0.003 | -0.15- -0.13 | <0.001 | -0.14 | 0.003 | -0.15- -0.13 | <.0001 |
| 50-64 | -0.06 | 0.004 | -0.07- -0.05 | <0.001 | -0.06 | 0.004 | -0.07- -0.05 | <.0001 |
| 65-74 | -0.03 | 0.004 | -0.04- -0.03 | <0.001 | -0.03 | 0.004 | -0.04- -0.03 | <.0001 |
| 75+ | -0.04 | 0.004 | -0.05- -0.03 | <0.001 | -0.04 | 0.004 | -0.05- -0.03 | <.0001 |
| Income Quintile |  |  |  |  |  |  |  |  |
| 1 | -0.01 | 0.002 | -0.009- -0.003 | <0.001 | -0.01 | 0.002 | -0.009- -0.0025 | 0.00 |
| 2 | -0.001 | 0.001 | -0.004- 0.001 | 0.24 | -0.001 | 0.001 | -0.004- 0.001 | 0.25 |
| 3 | -0.002 | 0.001 | -0.004- 0.0001 | 0.06 | -0.002 | 0.001 | -0.004- 0.0001 | 0.07 |
| 4 | -0.003 | 0.001 | -0.004- -0.001 | 0.01 | -0.003 | 0.001 | -0.004- -0.001 | 0.01 |
| 5 | Referent |  |  |  | Referent |  |  |  |
| Resource Utilization Band |  |  |  |  |  |  |  |  |
| 0 | Referent |  |  |  | Referent |  |  |  |
| 1 | -0.08 | 0.05 | -0.18- 0.03 | 0.16 | -0.08 | 0.05 | -0.18- 0.03 | 0.16 |
| 2 | -0.07 | 0.05 | -0.18- 0.03 | 0.18 | -0.07 | 0.05 | -0.18- 0.03 | 0.18 |
| 3 | -0.09 | 0.05 | -0.20- 0.02 | 0.10 | -0.09 | 0.05 | -0.20- 0.02 | 0.10 |
| 4 | -0.11 | 0.05 | -0.22- -0.01 | 0.04 | -0.11 | 0.05 | -0.22- -0.01 | 0.04 |
| 5 | -0.14 | 0.05 | -0.25- -0.04 | <0.01 | -0.14 | 0.05 | -0.25- -0.04 | 0.01 |
| Immigrant | 0.03 | 0.002 | 0.02- 0.03 | <0.001 | 0.03 | 0.002 | 0.02- 0.03 | <0.001 |
| Rurality |  |  |  |  |  |  |  |  |
| Urban | Referent |  |  |  | Referent |  |  |  |
| Suburban | 0.04 | 0.004 | 0.04- 0.05 | <0.001 | 0.04 | 0.004 | 0.04- 0.05 | <0.001 |
| Rural | 0.06 | 0.004 | 0.05- 0.06 | <0.001 | 0.06 | 0.004 | 0.05- 0.06 | <0.001 |
| Female Physician |  |  |  |  | -0.01 | 0.01 | -0.03- 0.01 | 0.35 |
| Physician Age |  |  |  |  | 0.004 | 0.0004 | 0.004- 0.01 | <0.001 |
| Foreign Trained |  |  |  |  | -0.05 | 0.01 | -0.07- -0.03 | <0.001 |
| Practice Group Size |  |  |  |  |  |  |  |  |
| 1 |  |  |  |  | Referent |  |  |  |
| 2-10 |  |  |  |  | 0.11 | 0.02 | 0.08- 0.14 | <0.001 |
| 11+ |  |  |  |  | 0.12 | 0.01 | 0.10- 0.15 | <0.001 |
| Model |  |  |  |  |  |  |  |  |
| FFS |  |  |  |  | Referent |  |  |  |
| Capitation, non-FHT |  |  |  |  | -0.004 | 0.02 | -0.04- 0.03 | 0.80 |
| Capitation FHT |  |  |  |  | -0.11 | 0.02 | -0.14- -0.08 | <.0001 |
| Panel Size |  |  |  |  | 0.0001 | 0.00 | 0.00- 0.0001 | <.0001 |

**DM- Eye Exam**

|  | Adjusted Patient Characteristics | | | | Fully Adjusted (Patient and Physician) | | | |
| --- | --- | --- | --- | --- | --- | --- | --- | --- |
|  | Estimate | Standard Deviation | 95% Confidence Limits | P-value | Estimate | Standard Deviation | 95% Confidence Limits | P-value |
| Female | 0.11 | 0.02 | 0.07- 0.15 | <0.001 | 0.10 | 0.02 | 0.06- 0.14 | <0.001 |
| Age Groups |  |  |  |  |  |  |  |  |
| 40-49 | Referent |  |  |  | Referent |  |  |  |
| 50-64 | 0.37 | 0.03 | 0.32- 0.43 | <0.001 | 0.38 | 0.03 | 0.32- 0.43 | <0.001 |
| 65-74 | 1.40 | 0.04 | 1.33- 1.48 | <0.001 | 1.41 | 0.04 | 1.33- 1.48 | <0.001 |
| 75+ | 1.31 | 0.04 | 1.23- 1.38 | <0.001 | 1.31 | 0.04 | 1.23- 1.39 | <0.001 |
| Income Quintile |  |  |  |  |  |  |  |  |
| 1 | -0.09 | 0.03 | -0.16- -0.02 | 0.01 | -0.08 | 0.03 | -0.15- -0.01 | 0.02 |
| 2 | 0.02 | 0.03 | -0.05- 0.08 | 0.61 | 0.02 | 0.03 | -0.05- 0.08 | 0.59 |
| 3 | -0.03 | 0.03 | -0.09- 0.03 | 0.29 | -0.03 | 0.03 | -0.09- 0.03 | 0.28 |
| 4 | -0.02 | 0.03 | -0.08- 0.04 | 0.51 | -0.02 | 0.03 | -0.08- 0.04 | 0.54 |
| 5 |  |  |  |  |  |  |  |  |
| Resource Utilization Band |  |  |  |  |  |  |  |  |
| 0 | Referent |  |  |  | Referent |  |  |  |
| 1 | 0.90 | 0.27 | 0.37- 1.44 | <0.001 | 0.93 | 0.28 | 0.38- 1.47 | <0.001 |
| 2 | 2.30 | 0.21 | 1.88- 2.72 | <0.001 | 2.33 | 0.22 | 1.90- 2.75 | <0.001 |
| 3 | 3.02 | 0.21 | 2.61- 3.43 | <0.001 | 3.05 | 0.22 | 2.62- 3.47 | <0.001 |
| 4 | 3.32 | 0.21 | 2.90- 3.73 | <0.001 | 3.34 | 0.22 | 2.91- 3.77 | <0.001 |
| 5 | 3.23 | 0.21 | 2.82- 3.65 | <0.001 | 3.26 | 0.22 | 2.83- 3.68 | <0.001 |
| Immigrant | -0.14 | 0.04 | -0.23- -0.05 | <0.01 | -0.15 | 0.04 | -0.23- -0.06 | <0.01 |
| Rurality |  |  |  |  |  |  |  |  |
| Urban | Referent |  |  |  | Referent |  |  |  |
| Suburban | 0.07 | 0.04 | -0.003 -0.14 | 0.06 | 0.07 | 0.04 | -0.0005- 0.14 | 0.05 |
| Rural | 0.11 | 0.04 | 0.03- 0.18 | <0.01 | 0.13 | 0.04 | 0.06- 0.20 | <0.001 |
| Female Physician |  |  |  |  | 0.07 | 0.03 | 0.01- 0.13 | 0.02 |
| Physician Age |  |  |  |  | -0.002 | 0.002 | -0.005- 0.001 | 0.22 |
| Foreign Trained |  |  |  |  | -0.07 | 0.04 | -0.14- 0.01 | 0.08 |
| Practice Group Size |  |  |  |  |  |  |  |  |
| 1 |  |  |  |  | Referent |  |  |  |
| 2-10 |  |  |  |  | 0.09 | 0.05 | -0.01- 0.19 | 0.08 |
| 11+ |  |  |  |  | 0.08 | 0.04 | -0.01- 0.16 | 0.07 |
| Model |  |  |  |  |  |  |  |  |
| FFS |  |  |  |  | Referent |  |  |  |
| Capitation, non-FHT |  |  |  |  | 0.05 | 0.05 | -0.05- 0.14 | 0.34 |
| Capitation FHT |  |  |  |  | 0.34 | 0.05 | 0.24- 0.45 | <0.001 |
| Panel Size |  |  |  |  | 0.00 | 0.00 | -0.0001- 0.00 | 0.13 |

**DM- HgA1c lab tests**

|  | Adjusted Patient Characteristics | | | | Fully Adjusted (Patient and Physician) | | | |
| --- | --- | --- | --- | --- | --- | --- | --- | --- |
|  | Estimate | Standard Deviation | 95% Confidence Limits | P-value | Estimate | Standard Deviation | 95% Confidence Limits | P-value |
| Female | -0.07 | 0.02 | -0.11- -0.04 | <0.001 | -0.08 | 0.02 | -0.11- -0.04 | <0.001 |
| Age Groups |  |  |  |  |  |  |  |  |
| 40-49 |  |  |  |  |  |  |  |  |
| 50-64 | 0.35 | 0.03 | 0.29- 0.42 | <0.001 | 0.36 | 0.03 | 0.29- 0.42 | <0.001 |
| 65-74 | 0.64 | 0.04 | 0.57- 0.71 | <0.001 | 0.65 | 0.04 | 0.58- 0.72 | <0.001 |
| 75+ | 0.54 | 0.04 | 0.47- 0.62 | <0.001 | 0.55 | 0.04 | 0.48- 0.63 | <0.001 |
| Income Quintile |  |  |  |  |  |  |  |  |
| 1 | 0.04 | 0.03 | -0.02- 0.09 | 0.22 | 0.04 | 0.03 | -0.02- 0.10 | 0.19 |
| 2 | 0.07 | 0.03 | 0.02- 0.12 | <0.01 | 0.08 | 0.03 | 0.02- 0.13 | <0.01 |
| 3 | 0.05 | 0.03 | -0.003- 0.10 | 0.06 | 0.05 | 0.03 | 0.00- 0.11 | 0.06 |
| 4 | 0.09 | 0.03 | 0.04- 0.14 | <0.001 | 0.09 | 0.03 | 0.04- 0.14 | <0.001 |
| 5 | Referent |  |  |  | Referent |  |  |  |
| Resource Utilization Band |  |  |  |  |  |  |  |  |
| ≤1 | Referent |  |  |  | Referent |  |  |  |
| 2 | 3.11 | 0.35 | 2.43- 3.79 | <0.001 | 3.20 | 0.38 | 2.46- 3.94 | <0.001 |
| 3 | 3.33 | 0.34 | 2.66- 4.00 | <0.001 | 3.42 | 0.38 | 2.69- 4.16 | <0.001 |
| 4 | 3.40 | 0.34 | 2.73- 4.08 | <0.001 | 3.50 | 0.37 | 2.76- 4.23 | <0.001 |
| 5 | 3.34 | 0.34 | 2.66- 4.01 | <0.001 | 3.43 | 0.37 | 2.69- 4.16 | <0.001 |
| Immigrant | -0.005 | 0.04 | -0.09- 0.08 | 0.91 | -0.01 | 0.04 | -0.09- 0.08 | 0.91 |
| Rurality |  |  |  |  |  |  |  |  |
| Urban | Referent |  |  |  | Referent |  |  |  |
| Suburban | -0.08 | 0.04 | -0.15- -0.0006 | <0.05 | -0.08 | 0.04 | -0.15- -0.0007 | 0.05 |
| Rural | -0.20 | 0.05 | -0.30- -0.09 | <0.001 | -0.19 | 0.05 | -0.30- -0.09 | 0.00 |
| Female Physician |  |  |  |  | 0.23 | 0.06 | 0.10- 0.35 | <0.001 |
| Physician Age |  |  |  |  | -0.01 | 0.003 | -0.01- 0.0001 | 0.05 |
| Foreign Trained |  |  |  |  | -0.17 | 0.08 | -0.32- -0.02 | 0.03 |
| Practice Group Size |  |  |  |  |  |  |  |  |
| 1 |  |  |  |  | Referent |  |  |  |
| 2-10 |  |  |  |  | 0.0006 | 0.11 | -0.21- 0.22 | 1.00 |
| 11+ |  |  |  |  | 0.10 | 0.09 | -0.08- 0.28 | 0.27 |
| Model |  |  |  |  |  |  |  |  |
| FFS |  |  |  |  | Referent |  |  |  |
| Capitation, non-FHT |  |  |  |  | 0.29 | 0.11 | 0.09- 0.5 | 0.01 |
| Capitation FHT |  |  |  |  | 0.08 | 0.10 | -0.12- 0.29 | 0.43 |
| Panel Size |  |  |  |  | 0.0001 | 0.00 | 0.00- 0.0002 | 0.18 |

**DM- Lipid Testing**

|  | Adjusted Patient Characteristics | | | | Fully Adjusted (Patient and Physician) | | | |
| --- | --- | --- | --- | --- | --- | --- | --- | --- |
|  | Estimate | Standard Deviation | 95% Confidence Limits | P-value | Estimate | Standard Deviation | 95% Confidence Limits | P-value |
| Female | -0.15 | 0.02 | -0.19- -0.12 | <0.001 | -0.16 | 0.02 | -0.20- -0.13 | <0.001 |
| Age Groups |  |  |  |  |  |  |  |  |
| 40-49 | Referent |  |  |  | Referent |  |  |  |
| 50-64 | 0.45 | 0.03 | 0.40- 0.51 | <0.001 | 0.46 | 0.03 | 0.40- 0.52 | <0.001 |
| 65-74 | 0.72 | 0.03 | 0.66- 0.78 | <0.001 | 0.73 | 0.03 | 0.67- 0.79 | <0.001 |
| 75+ | 0.31 | 0.03 | 0.25- 0.37 | <0.001 | 0.32 | 0.03 | 0.25- 0.38 | <0.001 |
| Income Quintile |  |  |  |  |  |  |  |  |
| 1 | -0.05 | 0.03 | -0.10- 0.005 | 0.07 | -0.05 | 0.03 | -0.10- 0.01 | 0.09 |
| 2 | 0.02 | 0.02 | -0.03- 0.07 | 0.49 | 0.02 | 0.03 | -0.03- 0.07 | 0.47 |
| 3 | -0.01 | 0.02 | -0.05- 0.04 | 0.81 | -0.01 | 0.02 | -0.05- 0.04 | 0.81 |
| 4 | 0.04 | 0.02 | -0.01- 0.08 | 0.15 | 0.04 | 0.02 | -0.01- 0.08 | 0.14 |
| 5 | Referent |  |  |  | Referent |  |  |  |
| Resource Utilization Band |  |  |  |  |  |  |  |  |
| 0 | Referent |  |  |  | Referent |  |  |  |
| 1 | 2.25 | 0.61 | 1.05- 3.46 | <0.001 | 2.35 | 0.70 | 0.97- 3.73 | <0.001 |
| 2 | 4.19 | 0.57 | 3.07- 5.31 | <0.001 | 4.33 | 0.66 | 3.04- 5.63 | <0.001 |
| 3 | 4.61 | 0.57 | 3.49- 5.73 | <0.001 | 4.76 | 0.66 | 3.46- 6.05 | <0.001 |
| 4 | 4.66 | 0.57 | 3.54- 5.78 | <0.001 | 4.81 | 0.66 | 3.51- 6.10 | <0.001 |
| 5 | 4.41 | 0.57 | 3.29- 5.53 | <0.001 | 4.55 | 0.66 | 3.26- 5.85 | <0.001 |
| Immigrant | -0.03 | 0.04 | -0.10- 0.05 | 0.50 | -0.03 | 0.04 | -0.10- 0.05 | 0.47 |
| Rurality |  |  |  |  |  |  |  |  |
| Urban | Referent |  |  |  | Referent |  |  |  |
| Suburban | -0.14 | 0.04 | -0.21- -0.06 | <0.001 | -0.12 | 0.04 | -0.2- -0.05 | <0.01 |
| Rural | -0.30 | 0.05 | -0.39- -0.21 | <0.001 | -0.28 | 0.05 | -0.37- -0.19 | <0.001 |
| Female Physician |  |  |  |  | 0.32 | 0.06 | 0.21- 0.44 | <0.001 |
| Physician Age |  |  |  |  | -0.01 | 0.003 | -0.02- -0.01 | <0.001 |
| Foreign Trained |  |  |  |  | 0.004 | 0.07 | -0.13- 0.14 | 0.96 |
| Practice Group Size |  |  |  |  |  |  |  |  |
| 1 |  |  |  |  | Referent |  |  |  |
| 2-10 |  |  |  |  | -0.03 | 0.10 | -0.23- 0.17 | 0.77 |
| 11+ |  |  |  |  | 0.18 | 0.08 | 0.02- 0.35 | 0.03 |
| Model |  |  |  |  |  |  |  |  |
| FFS |  |  |  |  | Referent |  |  |  |
| Capitation, non-FHT |  |  |  |  | -0.05 | 0.10 | -0.25- 0.15 | 0.60 |
| Capitation FHT |  |  |  |  | -0.11 | 0.10 | -0.30- 0.08 | 0.26 |
| Panel Size |  |  |  |  | 0.0001 | 0.00 | 0.00- 0.0002 | 0.07 |

**DM- ACE or ARB prescription**

|  | Adjusted Patient Characteristics | | | | Fully Adjusted (Patient and Physician) | | | |
| --- | --- | --- | --- | --- | --- | --- | --- | --- |
|  | Estimate | Standard Deviation | 95% Confidence Limits | P-value | Estimate | Standard Deviation | 95% Confidence Limits | P-value |
| Female | -0.12 | 0.02 | -0.16- -0.07 | <0.001 | -0.12 | 0.02 | -0.17- -0.07 | <.0001 |
| Age Groups |  |  |  |  |  |  |  |  |
| 65-74 | Referent |  |  |  |  |  |  |  |
| 75+ | -0.07 | 0.02 | -0.11- -0.02 | <0.01 | -0.06 | 0.02 | -0.11- -0.02 | 0.01 |
| Income Quintile |  |  |  |  |  |  |  |  |
| 1 | 0.08 | 0.04 | -0.005- 0.16 | 0.07 | 0.08 | 0.04 | 0.002- 0.16 | 0.05 |
| 2 | 0.06 | 0.04 | -0.01- 0.14 | 0.10 | 0.07 | 0.04 | -0.01- 0.14 | 0.09 |
| 3 | 0.05 | 0.04 | -0.02- 0.12 | 0.17 | 0.05 | 0.04 | -0.02- 0.12 | 0.17 |
| 4 | 0.06 | 0.04 | -0.02- 0.13 | 0.12 | 0.06 | 0.04 | -0.01- 0.13 | 0.11 |
| 5 | Referent |  |  |  | Referent |  |  |  |
| Resource Utilization Band |  |  |  |  |  |  |  |  |
| 0 | Referent |  |  |  | Referent |  |  |  |
| 1 | 0.90 | 0.55 | -0.18-1.99 | 0.10 | 0.92 | 0.57 | -0.19- 2.03 | 0.10 |
| 2 | 3.07 | 0.45 | 2.19-3.94 | <0.001 | 3.09 | 0.46 | 2.20- 3.99 | <.0001 |
| 3 | 3.31 | 0.44 | 2.45-4.18 | <0.001 | 3.33 | 0.45 | 2.45- 4.22 | <.0001 |
| 4 | 3.4 | 0.44 | 2.53-4.26 | <0.001 | 3.42 | 0.45 | 2.53- 4.30 | <.0001 |
| 5 | 3.44 | 0.44 | 2.57-4.31 | <0.001 | 3.46 | 0.45 | 2.57- 4.35 | <.0001 |
| Immigrant | -0.05 | 0.07 | -0.18-0.08 | 0.43 | -0.06 | 0.07 | -0.19- 0.07 | 0.36 |
| Rurality |  |  |  |  |  |  |  |  |
| Urban | Referent |  |  |  | Referent |  |  |  |
| Suburban | 0.11 | 0.04 | 0.03- 0.19 | <0.01 | 0.12 | 0.04 | 0.03- 0.20 | 0.01 |
| Rural | 0.18 | 0.05 | 0.08- 0.27 | <0.001 | 0.21 | 0.05 | 0.11- 0.30 | <.0001 |
| Female Physician |  |  |  |  | 0.10 | 0.04 | 0.02- 0.18 | 0.01 |
| Physician Age |  |  |  |  | -0.004 | 0.002 | -0.01- -0.0002 | 0.04 |
| Foreign Trained |  |  |  |  | 0.07 | 0.05 | -0.03- 0.17 | 0.16 |
| Practice Group Size |  |  |  |  |  |  |  |  |
| 1 |  |  |  |  | Referent |  |  |  |
| 2-10 |  |  |  |  | 0.10 | 0.06 | -0.03- 0.22 | 0.13 |
| 11+ |  |  |  |  | 0.06 | 0.06 | -0.05- 0.17 | 0.31 |
| Model |  |  |  |  |  |  |  |  |
| FFS |  |  |  |  | Referent |  |  |  |
| Capitation, non-FHT |  |  |  |  | 0.01 | 0.06 | -0.11- 0.13 | 0.89 |
| Capitation FHT |  |  |  |  | 0.28 | 0.06 | 0.15 -0.41 | <.0001 |
| Panel Size |  |  |  |  | 0.00 | 0.00 | 0.00- 0.0001 | 0.66 |

**DM- Lipid Lowering prescription**

|  | Adjusted Patient Characteristics | | | | Fully Adjusted (Patient and Physician) | | | |
| --- | --- | --- | --- | --- | --- | --- | --- | --- |
|  | Estimate | Standard Deviation | 95% Confidence Limits | P-value | Estimate | Standard Deviation | 95% Confidence Limits | P-value |
| Female | -0.24 | 0.02 | -0.29- -0.19 | <0.001 | -0.25 | 0.02 | -0.30- -0.20 | <0.001 |
| Age Groups |  |  |  |  |  |  |  |  |
| 65-74 | Referent |  |  |  | Referent |  |  |  |
| 75+ | -0.28 | 0.02 | -0.33- -0.23 | <0.001 | -0.28 | 0.02 | -0.32- -0.23 | <0.001 |
| Income Quintile |  |  |  |  |  |  |  |  |
| 1 | 0.05 | 0.04 | -0.02- 0.13 | 0.18 | 0.06 | 0.04 | -0.02- 0.13 | 0.13 |
| 2 | 0.09 | 0.04 | 0.02- 0.16 | 0.02 | 0.09 | 0.04 | 0.02- 0.16 | 0.02 |
| 3 | 0.10 | 0.04 | 0.02- 0.17 | 0.01 | 0.10 | 0.04 | 0.02- 0.17 | 0.02 |
| 4 | 0.03 | 0.04 | -0.04- 0.10 | 0.40 | 0.03 | 0.04 | -0.04- 0.10 | 0.39 |
| 5 | Referent |  |  |  | Referent |  |  |  |
| Resource Utilization Band |  |  |  |  |  |  |  |  |
| 0 | Referent |  |  |  | Referent |  |  |  |
| 1 | 0.17 | 0.57 | -0.94- 1.28 | 0.76 | 0.20 | 0.60 | -0.97- 1.37 | 0.74 |
| 2 | 2.43 | 0.36 | 1.72- 3.15 | <0.001 | 2.48 | 0.38 | 1.73- 3.23 | <0.001 |
| 3 | 2.74 | 0.36 | 2.04- 3.44 | <0.001 | 2.79 | 0.37 | 2.05- 3.52 | <0.001 |
| 4 | 2.87 | 0.36 | 2.17- 3.57 | <0.001 | 2.91 | 0.37 | 2.18- 3.65 | <0.001 |
| 5 | 2.99 | 0.35 | 2.29- 3.68 | <0.001 | 3.03 | 0.37 | 2.30- 3.76 | <0.001 |
| Immigrant | -0.18 | 0.06 | -0.29- -0.06 | <0.01 | -0.18 | 0.06 | -0.30- -0.07 | <0.01 |
| Rurality |  |  |  |  |  |  |  |  |
| Urban | Referent |  |  |  | Referent |  |  |  |
| Suburban | 0.01 | 0.04 | -0.08- 0.10 | 0.84 | 0.04 | 0.04 | -0.04- 0.13 | 0.32 |
| Rural | 0.09 | 0.05 | -0.01- 0.18 | 0.07 | 0.14 | 0.05 | 0.05- 0.23 | <0.01 |
| Female Physician |  |  |  |  | 0.18 | 0.04 | 0.10- 0.27 | <0.001 |
| Physician Age |  |  |  |  | -0.01 | 0.002 | -0.01- -0.003 | <0.01 |
| Foreign Trained |  |  |  |  | -0.10 | 0.05 | -0.21- 0.005 | 0.06 |
| Practice Group Size |  |  |  |  |  |  |  |  |
| 1 |  |  |  |  | Referent |  |  |  |
| 2-10 |  |  |  |  | 0.01 | 0.07 | -0.13- 0.15 | 0.93 |
| 11+ |  |  |  |  | 0.06 | 0.06 | -0.05- 0.18 | 0.28 |
| Model |  |  |  |  |  |  |  |  |
| FFS |  |  |  |  | Referent |  |  |  |
| Capitation, non-FHT |  |  |  |  | -0.11 | 0.07 | -0.24- 0.02 | 0.11 |
| Capitation FHT |  |  |  |  | 0.19 | 0.07 | 0.06- 0.33 | 0.01 |
| Panel Size |  |  |  |  | 0.00 | 0.00 | 0.00- 0.0001 | 0.35 |

**DM- Metformin**

|  | Adjusted Patient Characteristics | | | | Fully Adjusted (Patient and Physician) | | | |
| --- | --- | --- | --- | --- | --- | --- | --- | --- |
|  | Estimate | Standard Deviation | 95% Confidence Limits | P-value | Estimate | Standard Deviation | 95% Confidence Limits | P-value |
| Female | -0.02 | 0.14 | -0.29- 0.25 | 0.89 | -0.05 | 0.15 | -0.34- 0.23 | 0.73 |
| Age Groups |  |  |  |  |  |  |  |  |
| 65-74 | Referent |  |  |  | Referent |  |  |  |
| 75+ | -0.29 | 0.14 | -0.57- -0.01 | <0.05 | -0.28 | 0.14 | -0.56- 0.003 | 0.05 |
| Income Quintile |  |  |  |  |  |  |  |  |
| 1 | -0.05 | 0.24 | -0.52- 0.43 | 0.85 | -0.05 | 0.24 | -0.52- 0.43 | 0.84 |
| 2 | -0.27 | 0.22 | -0.71- 0.17 | 0.23 | -0.27 | 0.23 | -0.72- 0.17 | 0.23 |
| 3 | -0.24 | 0.21 | -0.65- 0.17 | 0.25 | -0.25 | 0.21 | -0.66- 0.16 | 0.23 |
| 4 | -0.12 | 0.22 | -0.56- 0.31 | 0.58 | -0.12 | 0.22 | -0.56- 0.32 | 0.59 |
| 5 | Referent |  |  |  | Referent |  |  |  |
| Resource Utilization Band |  |  |  |  |  |  |  |  |
| ≤2 | Referent |  |  |  | Referent |  |  |  |
| 3 | 0.84 | 0.35 | 0.16- 1.53 | 0.02 | 0.83 | 0.35 | 0.14- 1.53 | 0.02 |
| 4 | 1.05 | 0.36 | 0.34- 1.77 | <0.01 | 1.04 | 0.37 | 0.31- 1.76 | 0.01 |
| 5 | 0.60 | 0.36 | -0.11- 1.31 | 0.10 | 0.59 | 0.37 | -0.13- 1.31 | 0.11 |
| Immigrant | -1.3 | 0.18 | -1.65- -0.94 | <0.001 | -1.34 | 0.18 | -1.71- -0.98 | <0.001 |
| Rurality |  |  |  |  |  |  |  |  |
| Urban | Referent |  |  |  | Referent |  |  |  |
| Suburban | -0.34 | 0.19 | -0.71- 0.03 | 0.07 | -0.31 | 0.19 | -0.68- 0.07 | 0.11 |
| Rural | -0.14 | 0.18 | -0.50- 0.22 | 0.44 | -0.10 | 0.18 | -0.46- 0.26 | 0.58 |
| Female Physician |  |  |  |  | 0.10 | 0.18 | -0.25- 0.45 | 0.57 |
| Physician Age |  |  |  |  | -0.004 | 0.01 | -0.02- 0.01 | 0.62 |
| Foreign Trained |  |  |  |  | 0.14 | 0.20 | -0.25- 0.52 | 0.48 |
| Practice Group Size |  |  |  |  |  |  |  |  |
| 1 |  |  |  |  | Referent |  |  |  |
| 2-10 |  |  |  |  | 0.23 | 0.26 | -0.27- 0.74 | 0.37 |
| 11+ |  |  |  |  | 0.13 | 0.21 | -0.29- 0.56 | 0.53 |
| Model |  |  |  |  |  |  |  |  |
| FFS |  |  |  |  | Referent |  |  |  |
| Capitation, non-FHT |  |  |  |  | -0.25 | 0.26 | -0.75- 0.26 | 0.34 |
| Capitation FHT |  |  |  |  | -0.01 | 0.27 | -0.54- 0.51 | 0.96 |
| Panel Size |  |  |  |  | 0.00 | 0.0001 | -0.0002- 0.0002 | 0.97 |
